# Comparison of two molecular diagnostic methods for identifying Beijing genotype of *Mycobacterium tuberculosis*

**Published:** 2020-06

**Authors:** Ghorban Ali Mahghani, Mohammad Kargar, Farshid Kafilzadeh, Homa Davoodi, Ezzat Allah Ghaemi

**Affiliations:** 1 Department of Microbiology, Jahrom Branch, Islamic Azad University, Jahrom, Iran; 2 Infectious Diseases Research Center, Golestan University of Medical Sciences, Gorgan, Iran

**Keywords:** *Mycobacterium tuberculosis*, Real-time polymerase chain reaction, Beijing family

## Abstract

**Background and Objectives:**

The Beijing family of *Mycobacterium tuberculosis* has been identified as a severe pathogen among this species and found in many clinical isolates during the last decade. Early identification of such genotype is important for better prevention and treatment of tuberculosis. The present study performed to compare the efficiency of Real-Time PCR and IS*6110*-Based Inverse PCR methods to identify the Beijing family.

**Materials and Methods:**

This study was carried out on 173 clinical isolates of *Mycobacterium tuberculosis* complex in Golestan Province, northern Iran. DNA extraction performed by boiling and determining the Beijing and non-Beijing strains carried out using Real-Time PCR and IS*6110*-Based Inverse PCR.

**Results:**

In both Real-Time PCR and IS*6110*-Based Inverse PCR method, 24 specimens (13.9%) of the Beijing family were identified and the result of the IS*6110*-Based Inverse PCR method showed that all the Beijing strains in this region belonged to the Ancient Beijing sub-lineage.

**Conclusion:**

Although the efficacy of the two methods in the diagnosis of the Beijing family is similar, the IS*6110*-Based Inverse PCR is more applicable to the ability to detect new and old Beijing family.

## INTRODUCTION

Although the prevalence of tuberculosis has been declined in the world in recent years, but is still among the top 10 causes with a death toll of 1.3 million worldwide, ranking above HIV/AIDS in 2016 (1). There is 7 major *M. tuberculosis* lineage associated with specific geographic regions, that genetic differences between them affect the distribution and prevalence in different regions (2, 3). In addition, the pathogenicity, geographical distribution, antibiotic resistance and lack of response to treatment are also different (4–6). Determination of these genotypes can be an important method of understanding pathogenicity and control of tuberculosis in each region.

Strains of the Beijing family are responsible for many cases of tuberculosis epidemics and phylogenetically belong to second tuberculosis lineage (East-Asian). Firstly, it was reported in the northwest of China (7, 8). These strains have a higher virulence (9), antibiotic resistance (10, 11), rapid transmission ability (12) and relapse (13) than the other lineages and more prevalence across Asia and countries such as the Soviet Union and several other geographic regions such as North America (14, 15).

Several studies have shown that the isolates of Beijing family of *Mycobacterium tuberculosis* complex (MTBC) also have genetic heterogeneity (16, 17). Based on the presence or absence of IS*6110* insertion sequences in the noise of transfer function (NTF) area (18), this family is divided into old and modern sub-lineages. It has been shown that the severity and progression of the disease, the rate of transmission, antibiotic resistance and the geographical distribution of the new sub-lineage are greater than the old ones (16, 19, 20).

It is anticipated that modern sub-lineage would have a selective advantage over the old, which can be evaluated by examining differences in the characteristics of virulence. These sub-lineages, other than Korea and Japan, are the most prevalent in many other countries (17, 18, 20) and even in Japan are increasing rapidly (21).

Therefore, the aim of this study was to compare the efficiency of both Real-Time PCR and IS*6110*-Based Inverse PCR methods to identify the Beijing family.

## MATERIALS AND METHODS

### Sample collection of TB patients.

Pure colonies of 173 non-repetitive confirmed MTBC on Löwenstein–Jensen medium reconstituted. These MTBC isolates collected from tuberculosis patients in Golestan Province southeast of the Caspian Sea, during 2016 and diagnosed according to biochemical tests as mentioned previously by Babai et al. (22). In addition, the pure culture of *M. tuberculosis* H37RV and 14 Non-tuberculosis species of Mycobacteria (23) also used as positive and negative controls, respectively. This research has been approved by national committee for medical ethics (IR.GOUMS. REC.1396.230).

### Genomic DNA extraction.

Genomic DNA extracted by the boiling method. In brief, 2–3 pure colonies homogenized in sterile distilled water and heated to 80°C for 20 minutes, after centrifugation, the supernatant phase of solution used. For the quantitative evaluation of DNA, the OD values of the specimens were measured by a Spectrophotometer / Fluorometer (Ds-11FX + Denovix) and the purity was examined by electrophoresis (23).

### Determination of Beijing and non-Beijing families.

Beijing and non-Beijing families were determined according to the Hillemann method (24) using BJ and nBJ primers (for RV2820 and RV2819), in Real-Time PCR assay (Table 1). The Real-Time PCR reaction performed in 25 μl volume containing 12.5 μL ABI TaqMan Universal PCR Master Mix (Applied Biosystems, USA), 1 μL of each primer, 0.5 μL of the specific probe, 8.5 μL of distilled water, and 1.5 μL of the DNA template (15). After 15 minutes initial denaturation at 95°C, the Real-Time PCR reaction performed with 40 cycles and 35 cycles for Beijing and non-Beijing detection, respectively, by ABI 7300 Real-time PCR system (Foster City, CA, USA).

**Table 1.** TaqMan primers and probe used to detect Beijing from non-Beijing Using Real-Time PCR

**Primer (Name)**	**Sequence (5 to 3 )**	**Product Size (bp)**	**Specific fragment**	**Reference**
Beijing forward (BjF)	CTCGGCAGCTTCCTCGAT	129 bp	RV2820	24
Beijing reverse (BjR)	CGAACTCGAGGCTGCCTACTAC			
Fluorogenic probe (BjTM)	YAK-AACGCCAGAGACCAGCCGCCGGCT-DB			
Non-Beijing forward (nBjF)	AAGCATTCCCTTGACAGTCGAA	95 bp	RV2819	
Non-Beijing reverse (nBjR)	GGCGCATGACTCGAAAGAAG			

### Identification of the Beijing family by IS*6110-*based inverse PCR.

Identification of the Beijing family by IS*6110*-Based Inverse PCR was performed with Ris 1 and Ris 2 primers (Table 2) located outwardly at the 3′ and 5′ termini of IS*6110* according to the methods described by Mokrousov (25). The Tm values for these primers were 54 and 55°C, respectively, purified DNA sample (2 μl) was added to the PCR mixture (final volume, 30 μl) that contained 30 pmol of each primer, 4.6 mM MgCl_2_, 0.3 μl of *Taq* DNA polymerase and 0.6 μl concentrations of each dNTPs. The reaction performed in Eppendorf Mastercycler DNA Engine Thermal Cycler PCR under the following conditions: an initial denaturation at 96°C for 3 min, 30 cycles of denaturation 95°C for 1 min, annealing 56°C for 1 min, and elongation 72°C for 1 min and a final elongation 72°C for 4 min. The amplified fragments electrophoresed in 1.5% agarose gels and visualized under UV light. A control contamination with previously amplified amplicon was performed by including a negative control sample (distilled water) in each PCR run, no contamination was detected.

**Table 2.** Primers used to differentiate between old and new Beijing and non-Beijing family by IS*6110*-Based Inverse PCR

**IS*6110*-Based Inverse PCR primers are locared on outer side at the the end of IS*6110* 3′ and 5′**					

**Primers**	**Sequence**	**Size of product (bp)**					

		**Modern sub-family Beijing**	**Ancient sub-family Beijing**	**Non Beijing isolates**	**Non tuberculosis species**
Ris1 (forward)	5′-GGCTGAGGTCTCAGATCAG-3′	260	-	-	-
Ris2 (reverse)	5′-ACCCCATCCTTTCCAAGAAC-3′	290	290	-	-
		470	470	470	-

Beijing strains of *M. tuberculosis* were detected by analysis of the NTF region for the presence, number and orientation of IS*6110* insertions with two bundles (290, 470 bp) were designated as the “old” sub-lineage or three bundles (260, 290, 470 bp) were designated as “modern” sub-lineage within the Beijing genotype. The single bundle of 470 bp regarded as non-Beijing (26).

### Statistical methods.

Statistical analysis performed to compare the frequency of Beijing and non-Beijing families based on demographic factors using analysis of variance in SPSS-22 software.

## RESULTS

### Identification of Beijing family by two methods.

The frequency of Beijing family among 173 MTB isolates in both Real-Time PCR and IS*6110*-Based Inverse PCR methods was 24 (13.9%). In addition, both methods have consistency in the determination of 14 non-tuberculosis isolates. Table 3 showed the frequency of Beijing and non-Beijing sub-lineages according to demographic criteria. Among them, only the mean age of patients (P = 0.002) between two group showed a statistically significant difference.

**Table 3.** Frequency of Beijing and non-Beijing sub-lineages based on demographic factors in tuberculosis patients in Golestan Province 2016

**Characteristic**		**Total**	**Beijing**	**Non-Beijing**	**P-value**
No. of patients (%)		173 (100)	24 (13.9)	149 (86.1)	0.408
Ethnicity	Fars	95 (54.92)	12 (12.6)	83 (87.4)	
	Baluch and Sistani	50 (28.91)	10 (20)	40 (80)	
	Torkman	25 (14.45)	2 (8)	25 (92)	
	Other	3 (1.74)	0 (0)	3 (100)	
Habitat	Rural	93 (53.76)	16 (17.2)	77 (82.8)	0.125
	Urban	80 (46.24)	8 (10)	72 (90)	
Sex	Male	85 (49.13)	14 (16.5)	71 (83.5)	0.226
	Female	88 (50.87)	10 (11.4)	78 (88.6)	
Mean Age(year)		49.6 ± 20.71	36.5 ± 20.61	51.7 ± 20.00	0.002^*^

* The numerator shows the number of cases of Beijing and denominator the number of positive culture *M. tuberculosis* in each city.

**Fig. 1.**
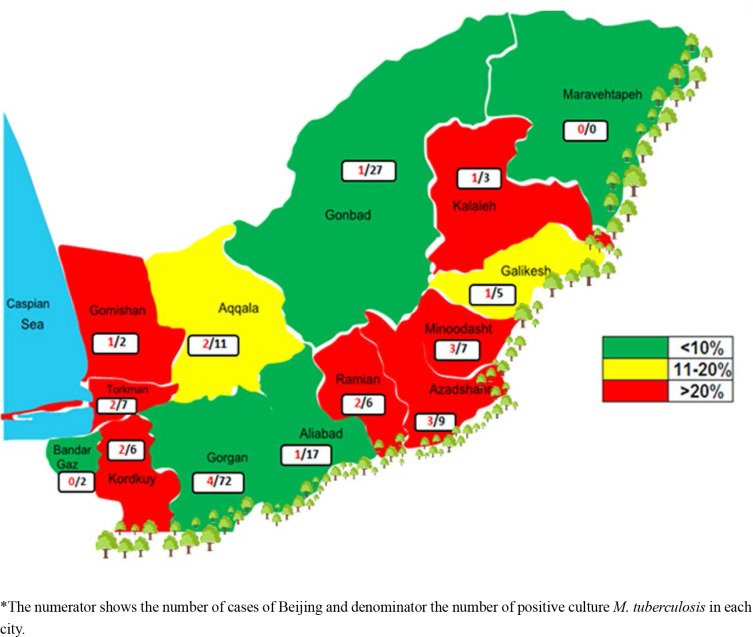
Geographical distribution of Beijing sub-lineage *M. tuberculosis* in Golestan Province, north of Iran

As shown in Fig. 1, Beijing sub-lineage does not have uniform distribution in different parts of the Golestan Province, as its incidence in regions on the margin of the Caspian Sea and the margin of the forest are higher than other parts.

**Fig. 2.**
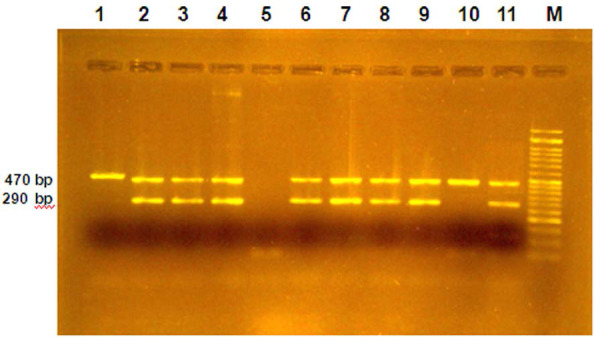
Results of gel electrophoresis with a concentration of 1.5% for the product of IS*6110*-based inverse PCR.

The results of the study using IS*6110*-Based Inverse PCR gel electrophoresis showed that all strains of Beijing in Golestan province were related to the ancient sub-lineage, with two bundles of 470 and 290 bp, and no case of new sub-lineage was found (Fig. 2). Modern Beijing sub-lineage strains create three bundles of 260, 290, 470 bp on the gel that has not been observed in Beijing isolates of Golestan Province.

The old Beijing Sub-lineage has two bundles, 470 and 290 bp (lane 2–4 and 6–9 and 11). Lane 5 negative control (non-tuberculosis strain) and lane 1 and 10 include *M. tuberculosis* H37Rv (from Tuberculosis Reference Laboratory of Golestan Province, Gorgan, Iran) in culture and a non-Beijing strain, Lane M is the DNA marker 50 bp.

## DISCUSSION

In this study, two methods of Real-Time PCR and IS*6110*-Based Inverse PCR used to study the prevalence of Beijing family in Golestan Province, northeastern Iran, which had the same results. Based on the present comparative study, both methods are accurate enough to determine the frequency of Beijing strains, but the IS*6110*-Based Inverse PCR method is easy to perform and does not require complex equipment and techniques. Moreover, low cost, time-consuming and the possibility of differentiation between old and new Beijing sub-lineages made it a good technique for rapid epidemiological studies.

Several studies conducted to determine the prevalence of Beijing in Iran and the world. The Beijing family of MTBC has its origin in China where it is the dominant type of *M. tuberculosis* but it has also shown to have a global distribution (14). It comprises about 50% of the TB species in East Asia and 13% of global isolates (27). In Iran, the frequency of Beijing family in different areas varies from 3.2% to 20.5% with average of 6.8% (15, 26, 28–34). In the northwestern and western Provinces of the country, prevalence has been estimated at 9–10% (28, 31, 35) in the Khorasan Province, located in the east of Iran, 7.1% (31), and in Tehran, the capital of Iran, 5.0% (29) and 13.9% in Golestan Province (15). On the other hand, these data confirm that the Beijing family abundance in Golestan province has remained unchanged for 5 years (2011–2016).

All strains of the Beijing genotype isolated in Golestan Province belong to old Beijing sub-lineage, which is consistent with the results of Mirbagheri et al. (26) in north-east of Iran in 2016, but in the most countries, apart from Japan, modern Beijing strains are more prevalent than the old strains (20, 21).

Old and modern Beijing strains are genetically closely related (36) but have been reported to possess some significant pathogenic properties such as differences in drug resistance, and the ability to cause disease and spread (12).

## CONCLUSION

Beijing family is an important sub-lineage of tuberculosis in the southeast of the Caspian Sea, especially in younger tuberculosis patients that can be similarly diagnosed by both Real-Time PCR and IS*6110*-Based Inverse PCR method. IS*6110*-Based Inverse PCR method is a preferred method because of its ability to the differentiation between new and old Beijing isolates.

## References

[1] World Health Organization. Global tuberculosis report 2013. World Heal Organ. 2013 (Appia, Geneva, Switzerland: WHO Press). Available from: https://apps.who.int/iris/handle/10665/91355

[2] Comas I Coscolla M Luo T Borrell S Holt KE Kato-Maeda M Out-of-Africa migration and Neolithic coexpansion of *Mycobacterium tuberculosis* with modern humans. Nat Genet 2013;45: 1176–1182. 23995134 10.1038/ng.2744 PMC3800747

[3] Firdessa R Berg S Hailu E Schelling E Gumi B Erenso G Mycobacterial lineages causing pulmonary and extrapulmonary tuberculosis, Ethiopia. Emerg Infect Dis 2013;19: 460–463. 23622814 10.3201/eid1903.120256 PMC3647644

[4] Arif HM Hussain Z. Prevalence of *Mycobacterium tuberculosis* Beijing strains in Punjab Pakistan. Int J Curr Res Aca Rev 2014;2:74–82.

[5] Coll F Phelan J Hill-Cawthorne GA Nair MB Mallard K Ali S Genome-wide analysis of multi-and extensively drug-resistant *Mycobacterium tuberculosis*. Nat Genet 2018;50:307–316. 29358649 10.1038/s41588-017-0029-0

[6] Roycroft E O’Toole R Fitzgibbon M Montgomery L O’Meara M Downes P *Molecular epidemiology* of multi-and extensively-drug-resistant *Mycobacterium tuberculosis* in Ireland, 2001–2014. J Infect 2018;76:55–67. 29031637 10.1016/j.jinf.2017.10.002

[7] Bainomugisa A Duarte T Lavu E Pandey S Coulter C Marais B A complete nanonpore-only assembly of an XDR *Mycobacterium tuberculosis* Beijing lineage strain identifies novel genetic variation in repetitive PE/PPE gene regions. Microbial genom 2018; 4. doi: 10.1099/mgen.0.000188. PMC6113869 29906261

[8] Mokrousov I Chernyaeva E Vyazovaya A Skiba Y Solovieva N Valcheva V Rapid assay for detection of the epidemiologically important central Asian/ Russian strain of the *Mycobacterium tuberculosis* Beijing genotype. J Clin Microbiol 2018;56(2):e01551–17. 29142045 10.1128/JCM.01551-17 PMC5786733

[9] Lasunskaia E Ribeiro SC Manicheva O Gomes LL Suffys PN Mokrousov I Emerging multidrug resistant *Mycobacterium tuberculosis* strains of the Beijing genotype circulating in Russia express a pattern of biological properties associated with enhanced virulence. Microbes Infect 2010;12:467–75. 20215000 10.1016/j.micinf.2010.02.008

[10] Buu TN Huyen M Lan N Quy H Hen N Zignol M The Beijing genotype is associated with young age and multidrug-resistant tuberculosis in rural Vietnam. Int J Tuberc Lung Dis 2009;13:900–906. 19555542

[11] Pasechnik O Vyazovaya A Vitriv S Tatarintseva M Blokh A Stasenko V Major genotype families and epidemic clones of *Mycobacterium tuberculosis* in Omsk region, Western Siberia, Russia, marked by a high burden of tuberculosis-HIV coinfection. Tuberculosis 2018;108:163–168. 29523319 10.1016/j.tube.2017.12.003

[12] Hanekom M Van Der Spuy G Streicher E Ndabambi S McEvoy C Kidd M A recently evolved sublineage of the *Mycobacterium tuberculosis* Beijing strain family is associated with an increased ability to spread and cause disease. J Clin Microbiol 2007;45:1483–1490. 17360841 10.1128/JCM.02191-06 PMC1865897

[13] Huyen MN Buu TN Tiemersma E Lan NT Dung NH Kremer K Tuberculosis relapse in Vietnam is significantly associated with *Mycobacterium tuberculosis* Beijing genotype infections. J Infect Dis 2013;207:1516–24. 23408848 10.1093/infdis/jit048

[14] Bifani PJ Mathema B Kurepina NE Kreiswirth BN. Global dissemination of the *Mycobacterium tuberculosis* W-Beijing family strains. Trends Microbiol 2002;10:45–52. 11755085 10.1016/s0966-842x(01)02277-6

[15] Erie H Kaboosi H Javid N Shirzad-Aski H Taziki M Kuchaksaraee MB The high prevalence of *Mycobacterium tuberculosis* Beijing strain at an early age and extra-pulmonary tuberculosis cases. Iran J Microbiol 2017;9:312–317. 29487728 PMC5825930

[16] Ribeiro SC Gomes LL Amaral EP Andrade MR Almeida FM Rezende AL *Mycobacterium tuberculosis* strains of the modern sublineage of the Beijing family are more likely to display increased virulence than strains of the ancient sublineage. J Clin Microbiol 2014;52:2615–2624. 24829250 10.1128/JCM.00498-14 PMC4097719

[17] Merker M Blin C Mona S Duforet-Frebourg N Lecher S Willery E Evolutionary history and global spread of the *Mycobacterium tuberculosis* Beijing lineage. Nat Genet 2015;47:242–249. 25599400 10.1038/ng.3195 PMC11044984

[18] Devi KR Bhutia R Bhowmick S Mukherjee K Mahanta J Narain K. Genetic diversity of *Mycobacterium tuberculosis* isolates from Assam, India: dominance of Beijing family and discovery of two new clades related to CAS1_Delhi and EAI family based on spoligotyping and MIRU-VNTR typing. PLoS One 2015;10(12): e0145860. 26701129 10.1371/journal.pone.0145860 PMC4689458

[19] Chakraborty P Kulkarni S Rajan R Sainis K. *Mycobacterium tuberculosis* strains from ancient and modern lineages induce distinct patterns of immune responses. J Infect Dev Ctries 2018;11:904–911. 31626595 10.3855/jidc.8596

[20] Liu Q Luo T Dong X Sun G Liu Z Gan M Genetic features of *Mycobacterium tuberculosis* modern Beijing sublineage. Emerg Microbes Infect 2016;5(2):e14. 26905026 10.1038/emi.2016.14 PMC4777927

[21] Iwamoto T Fujiyama R Yoshida S Wada T Shirai C Kawakami Y. Population structure dynamics of *Mycobacterium tuberculosis* Beijing strains during past decades in Japan. J Clin Microbiol 2009;47:3340–3343. 19710282 10.1128/JCM.01061-09 PMC2756919

[22] Kochkaksaraei MB Kaboosi H Ghaemi EA. Genetic variation of the *Mycobacterium tuberculosis* in north of Iran; the Golestan Province. Iran Red Crescent Med J 2019; 21 (8); e91553.

[23] Shafipour M Ghane M Alang SR Livani S Javid N Shakeri F. Non tuberculosis Mycobacteria isolated from tuberculosis patients in Golestan province, North of Iran. Ann Biol Res 2013;4:133–137.

[24] Hillemann D Warren R Kubica T Rüsch-Gerdes S Niemann S. Rapid detection of *Mycobacterium tuberculosis* Beijing genotype strains by real-time PCR. J Clin Microbiol 2006;44:302–306. 16455874 10.1128/JCM.44.2.302-306.2006 PMC1392668

[25] Mokrousov I Jiao WW Valcheva V Vyazovaya A Otten T Ly HM Rapid detection of the *Mycobacterium tuberculosis* Beijing genotype and its ancient and modern sublineages by IS*6110*-Based Inverse PCR. J Clin Microbiol 2006;44:2851–2856. 16891502 10.1128/JCM.00705-06 PMC1594662

[26] Mirbagheri SZ Meshkat Z Naderinasab M Ghadamsoltani T Rostami S Heravi MM Frequency of Beijing family of *Mycobacterium tuberculosis* in Mashhad, Northeast of Iran. Arch Med Lab Sci 2016;2:102–107.

[27] Hoffner S Sahebi L Ansarin K Sabour S Mohajeri P. *Mycobacterium tuberculosis* of the Beijing genotype in Iran and the World Health Organization Eastern Mediterranean Region: a meta-analysis. Microb Drug Resist 2018;24:693–698. 29058526 10.1089/mdr.2017.0160

[28] Doroudchi M Kremer K Basiri EA Kadivar MR Van Soolingen D Ghaderi AA. IS*6110*-RFLP and spoligo-typing of *Mycobacterium tuberculosis* isolates in Iran. Scand J Infect Dis 2000;32:663–8. 11200378 10.1080/003655400459595

[29] Farnia P Masjedi MR Mirsaeidi M Mohammadi F Vincent V Bahadori M Prevalence of Haarlem I and Beijing types of *Mycobacterium tuberculosis* strains in Iranian and Afghan MDR-TB patients. J Infect 2006;53:331–336. 16476483 10.1016/j.jinf.2005.12.020

[30] Velayati AA Farnia P Mirsaeidi M Reza Masjedi M. The most prevalent *Mycobacterium tuberculosis* superfamilies among Iranian and Afghan TB cases. Scand J Infect Dis 2006;38:463–468. 16798695 10.1080/00365540500504117

[31] Rohani M Farnia P Nasab MN Moniri R Torfeh M Amiri M. Beijing genotype and other predominant *Mycobacterium tuberculosis* spoligotypes observed in Mashhad city, Iran. Indian J Med Microbiol 2009;27:306–310. 19736398 10.4103/0255-0857.55441

[32] Kazempour M Masjedi M Velayati A Tajeddin E Farnia P Kargar M Identification of *Mycobacterium tuberculosis* beijing genotype using three different molecular methods. Koomesh 2009;11:7–14.

[33] Mozafari M Farnia P Jafarian M Razavi Deligani M Kazempour M Masjedi M Comparison of *Mycobacterium tuberculosis* Beijing genotype with other Mycobacterium tuberculosis strains Using MIRU-VNTR method. Iran South Med J 2012;15:1–12.

[34] Khosravi AD Goodarzi H Alavi SM Akhond MR. Application of deletion-targeted multiplex PCR technique for detection of *Mycobacterium tuberculosis* Beijing strains in samples from tuberculosis patients. Iran J Microbiol 2014;6: 330–334. 25848523 PMC4385573

[35] Mohajeri P Moradi S Atashi S Farahani A. *Mycobacterium tuberculosis* Beijing genotype in western Iran: Distribution and drug resistance. Journal of clinical and diagnostic research. J Clin Diagn Res 2016;10: DC05–DC07. 10.7860/JCDR/2016/20893.8689 PMC5121674 27891336

[36] Schürch AC Kremer K Warren RM Hung NV Zhao Y Wan K Mutations in the regulatory network underlie the recent clonal expansion of a dominant sub-clone of the *Mycobacterium tuberculosis* Beijing genotype. Infect Genet Evol 2011;11:587–597. 21277396 10.1016/j.meegid.2011.01.009

